# Long-term sustainability and safety of a delivery-based early-onset sepsis evaluation strategy for very low birth weight infants

**DOI:** 10.1038/s41372-026-02607-y

**Published:** 2026-03-13

**Authors:** Molly F. May, Alvaro Zevallos Barboza, Samuel J. Garber, Karen M. Puopolo, Sagori Mukhopadhyay, Dustin D. Flannery

**Affiliations:** 1 Division of Neonatology, Children’s Hospital of Philadelphia, Philadelphia, PA USA; 2 University of Pennsylvania School of Nursing, Philadelphia, PA USA; 3 Department of Pediatrics, University of Pennsylvania Perelman School of Medicine, Philadelphia, PA USA

**Keywords:** Bacterial infection, Paediatrics

## Introduction

Empiric antibiotics are given to about 80% of very low birth weight (VLBW) infants in the United States for concern of early-onset sepsis (EOS) [1]. We previously reported that implementing a delivery-based risk-stratification guideline for EOS evaluation among preterm infants in1 our level III neonatal intensive care unit (NICU), consistent with American Academy of Pediatrics (AAP) recommendations, was associated with reduced early antibiotic use without safety concerns [2, 3].

Infants are classified as low-risk if born by cesarean delivery for maternal indications (e.g., preeclampsia), without labor or attempted induction of labor, and with rupture of membranes at delivery [2, 4, 5]. In this study, we assessed the sustainability and safety of this guideline over a four-year period following its initial implementation.

## Methods

We conducted a retrospective cohort study of VLBW infants (<1500 g) admitted to the NICU from January 2009 to April 2025. The study was approved by the University of Pennsylvania IRB.

Three study periods were defined: pre-implementation (January 2009–March 2017), post-implementation (April 2017–January 2020), and sustainability (February 2020–April 2025). Clinical data were extracted from the electronic medical record. The primary outcome was the proportion of VLBW infants (overall and low-risk) with antibiotic initiation on days 0–3 after birth. Balancing measures included antibiotic initiation on days 4–7, blood or spinal fluid culture-confirmed infection on days 4–7, and death or transfer by day 7.

Comparisons were made across periods using descriptive statistics. Annual rates of antibiotic initiation among low-risk VLBW infants were displayed graphically.

## Results

The study included 1253 VLBW infants: 727 during pre-implementation, 191 during post-implementation, and 335 during the sustainability period (Supplementary Table 1). Baseline characteristics were similar across cohorts. The proportion classified as low-risk was 41%, 44%, and 35% across the three periods.

**Fig. 1:**
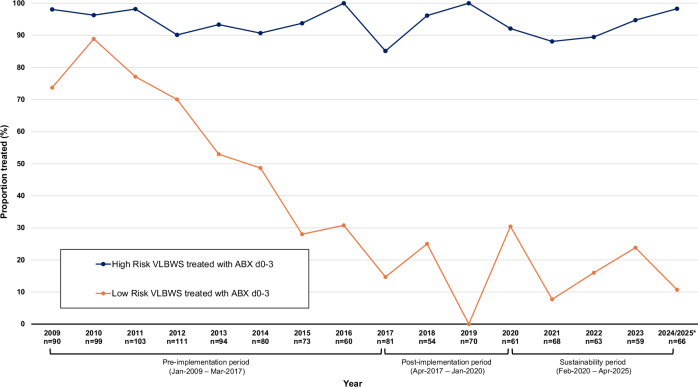
Annual rates of early antibiotic initiation among very low birth weight infants by risk category, 2009–2025. Annual proportions of very low birth weight (VLBW) infants receiving antibiotics on days 0–3 after birth from 2009 through April 2025 are shown by delivery risk category. The orange line represents low-risk infants (delivered by cesarean for maternal indications, without labor or rupture of membranes before delivery), and the blue line represents higher-risk infants. * Data for 2024–2025 were combined, as the sustainability period includes infants born through April 2025 only.

Among low-risk infants, early antibiotic initiation decreased from 185/298 (62.1%) pre-implementation to 11/83 (13.3%) post-implementation and remained low in the sustainability period at 21/122 (17.2%), with no significant difference between post-implementation and sustainability (*p* = 0.4, Supplementary Table 2). For days 4–7, rates of blood culture collection, antibiotic initiation, bacteremia, and death or transfer were similar across periods. As rates of early antibiotic initiation among low-risk infants declined, the rate among high-risk infants was unchanged over the study period (Fig. 1).

To assess whether changes were limited to low-risk infants or reflected broader practice shifts, we compared antibiotic use among all VLBW infants. Antibiotic initiation increased in the sustainability period compared to post-implementation (113/191 [59.2%] vs. 221/335 [66.0%], *p* = 0.04), though the proportion of low-risk infants was lower (43.5% vs. 36.4%, *p* = 0.05). During days 4–7, both antibiotic initiation (11.5% vs. 6%, *p* = 0.03) and blood culture collection (13.1% vs. 7.2%, *p* = 0.02) were lower in the sustainability period. Rates of death, transfer, and culture-confirmed EOS were similar across periods. Overall, 18/738 (2.4%) high-risk infants had culture-confirmed EOS, while no cases occurred among 488 low-risk infants (*p* < 0.001).

During the sustainability period, 21 low-risk infants (17.2%) received early antibiotics. Documented reasons included clinical change (*n* = 10), hypotension (*n* = 5), gastrointestinal pathology (*n* = 4), poor biophysical profile (*n* = 1), and hyperglycemia (*n* = 1).

## Discussion

Implementation of a delivery-based EOS risk-stratification guideline based on AAP recommendations for VLBW infants was sustained for over seven years without safety concerns. Early antibiotic initiation among low-risk infants remained stable and low through the sustainability period, and no cases of culture-confirmed EOS occurred in this group at any time during the study. The continued reduction in antibiotic initiation and blood culture collection during days 4–7 suggests growing provider confidence in the guideline.

These findings build on prior work demonstrating that delivery-based risk stratification can reduce the use of early empiric antibiotics in preterm infants [3–5]. Sustained reductions indicate that this approach is durable and adaptable within a high-volume NICU. Delivery-based criteria are simple, reproducible, and rely on routine obstetric data. Broader adoption may help reduce antibiotic exposure in very preterm infants, where empiric use remains common [1]. Further, adoption of this approach helps clinicians recognize not only those VLBW at very low risk of EOS, but also highlights the characteristics of VLBW infants at highest risk of EOS, a potentially fatal complication of preterm birth.

## Conclusion

Sustained reductions in early empiric antibiotics among VLBW infants highlight the feasibility of risk-stratified stewardship as a long-term practice standard.

## Supplementary information

Supplement

## Data Availability

De-identified data are available from the corresponding author upon reasonable request.
