# Supplementary material for: Long-term sustainability and safety of a delivery-based early-onset sepsis evaluation strategy for very low birth weight infants
Source: J Perinatol. 2026 Mar 13;46(5):865–7. doi: 10.1038/s41372-026-02607-y (PMC13190322; doi:10.1038/s41372-026-02607-y)
Supplement: Supplementary file 1 — Supplement [file 41372_2026_2607_MOESM1_ESM.docx]

**Supplementary Table 1: Baseline characteristics of very low birth weight (VLBW) infants across the three study periods (pre-implementation, post-implementation, and sustainability).**

|  | **Pre-implementation Period 1**  **n=727** | **Post-implementation Period 2**  **n=191** | **Sustainability Period 3**  **n=335** | **p-value** | |
| --- | --- | --- | --- | --- | --- |
|  |  |  |  | **Period 1 vs. 2^&^** | **Period 2 vs. 3** |
| Birth weight (grams), median (IQR)^*^ | 1085 (820 – 1320) | 1105 (795 – 1335) | 1085 (805-1355) | 0.74 | 0.77 |
| Gestational age (weeks), median (IQR)^*^ | 28 4/7  (26 3/7, 30 4/7) | 28 5/7  (26 3/7, 30 4/7) | 28 5/7  (263/7, 31 0/7) | 0.98 | 0.43 |
| Female sex, n (%) | 365 (50.2) | 107 (56.0) | 161 (48.1) | 0.15 | 0.37 |
| Multiple gestation, n (%) | 233 (32.0) | 41 (21.5) | 88 (26.3) | 0.004 | 0.22 |
| Cesarean delivery, n (%) | 564 (77.6) | 137 (71.7) | 242 (72.2) | 0.09 | 0.90 |
| Rupture of membranes at delivery, n (%) | 419 (57.6) | 128 (67.0) | 215 (64.2) | 0.02 | 0.51 |
| Infants meeting low-risk criteria, n (%) | 298 (41.0) | 83 (43.5) | 122 (36.4) | 0.54 | 0.11 |

**Footnotes**: ^&^Data from Garber, *et al. J Perinatol* 2021.

^*^Continuous variables were analyzed using the Mann-Whitney U test to assess differences between groups, given the non-normal distribution of the data.

**Supplementary Table 2: Clinical outcomes of VLBW infants, including low-risk subgroups, during the three study periods (pre-implementation, post-implementation, and sustainability).**

|  | **All VLBW infants** | | | **p-value** | | **Low-Risk VLBW Infants** | | | **p-value** | |
| --- | --- | --- | --- | --- | --- | --- | --- | --- | --- | --- |
|  | **Pre-implementation Period 1**  **n=727** | **Post- implementation Period 2**  **n=191** | **Sustainability Period 3**  **n=335** | **Period 1 vs. 2^&^** | **Period 2 vs. 3** | **Pre-implementation Period 1**  **n=298** | **Post- implementation Period 2**  **n=83** | **Sustainability Period 3**  **n=122** | **Period 1**  **vs. 2^&^** | **Period 2**  **vs. 3** |
| **Day 0-3 after birth, n (%)** | | | | | | | | | | |
| Blood culture obtained, n (%) | 643 (88.5) | 117 (61.3) | 221 (66.0) | <0.001 | 0.28 | 225 (75.5) | 14 (16.9) | 21 (17.2) | <0.001 | 0.95 |
| Antibiotic initiation, n (%) | 590 (81.2) | 113 (59.2) | 221  (66.0) | <0.001 | 0.12 | 185 (62.1) | 11 (13.3) | 21 (17.2) | <0.001 | 0.44 |
| Blood culture positive for a pathogen, n (%) | 9 (1.2) | 3 (1.6) | 6 (1.8) | 0.72 | 1.0* | 0 | 0 | 0 | - | - |
| **Day 4-7 after birth, n (%)** | | | | | | | | | | |
| Blood culture obtained, n (%) | 130 (17.9) | 25 (13.1) | 24 (7.2) | 0.12 | 0.02 | 61 (20.5) | 9 (10.8) | 10 (8.2) | 0.05 | 0.52 |
| Antibiotic initiation, n (%) | 67 (9.2) | 22 (11.5) | 22 (6.6) | 0.34 | 0.05 | 34 (11.4) | 9 (10.8) | 8 (6.6) | 0.89 | 0.27 |
| Blood culture positive for a pathogen, n (%) | 17 (2.3) | 3 (1.6) | 3 (0.9) | 0.78* | 0.67* | 6 (2.0) | 1 (1.2) | 0 | 1.0 * | 0.43* |
| Deceased/Transferred by 7 days age, n (%) | 36 (5.0) | 9 (4.7) | 12(3.6) | 0.89 | 0.52 | 11 (3.7) | 2 (2.4) | 1 (0.8) | 0.74* | 0.57* |

**Footnote**: ^&^Data from Garber, et al*. J Perinatol* 2021.

^*^ P-values were calculated using Fisher's exact test to ensure accuracy with small sample sizes and low expected frequencies.
